# Supplementary figures and images for: Global Transcriptomic Response of Staphylococcus aureus to Virulent Bacteriophage Infection
Source: Viruses. 2022 Mar 9;14(3):567. doi: 10.3390/v14030567 (PMC8950790; doi:10.3390/v14030567)

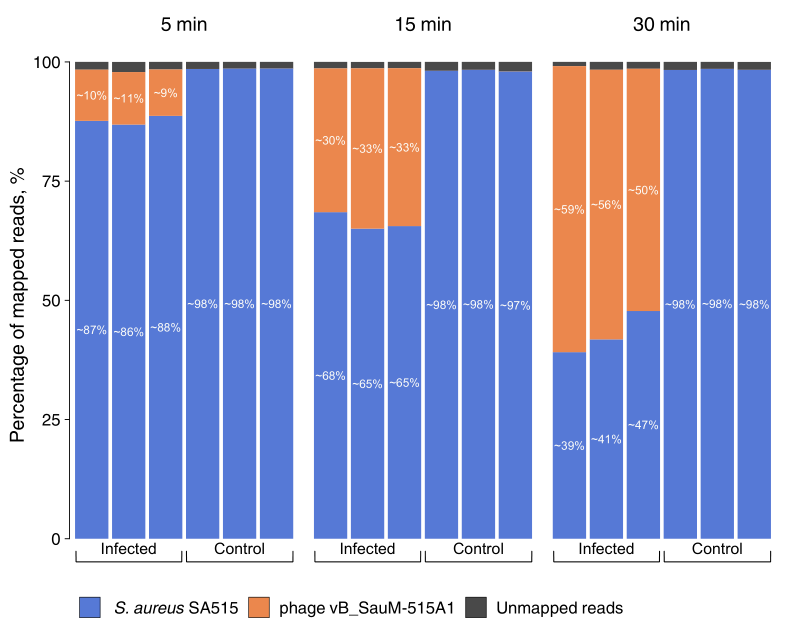

Supplement: Supplementary file 1 [file viruses-14-00567-s001.zip › Figure S1.tiff]

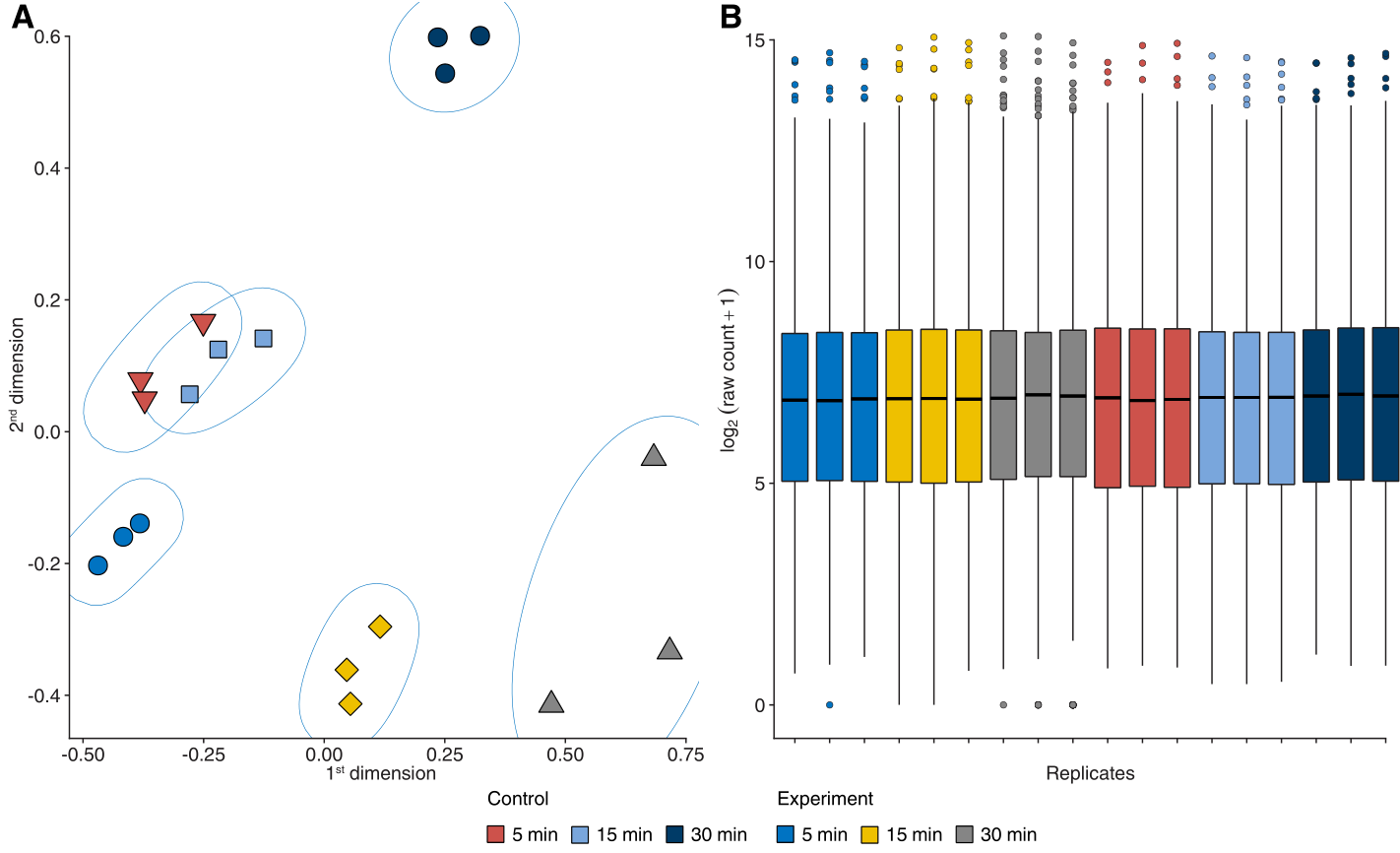

Supplement: Supplementary file 1 [file viruses-14-00567-s001.zip › Figure S2.tiff]

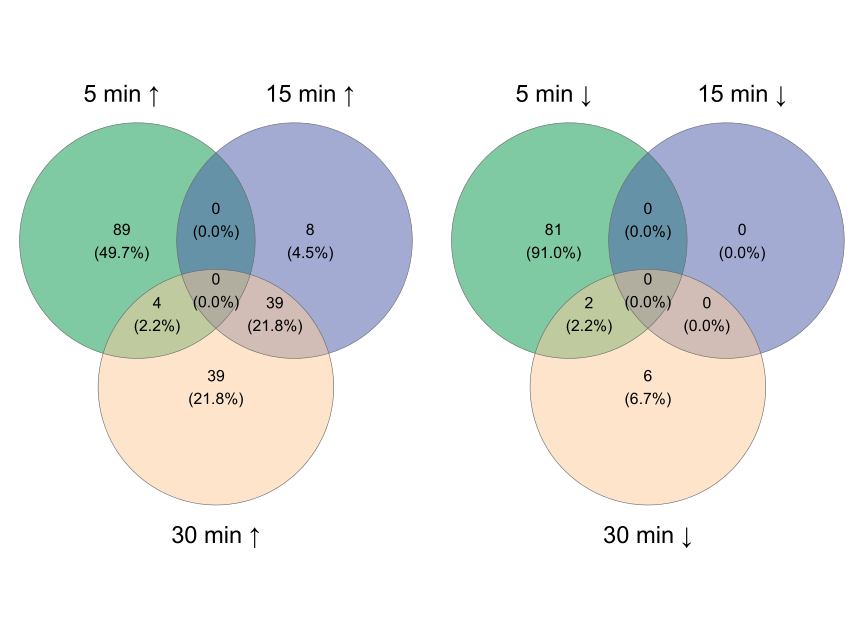

Supplement: Supplementary file 1 [file viruses-14-00567-s001.zip › Figure S3.tiff]
